# The geographic scale of diversification on islands: genetic and morphological divergence at a very small spatial scale in the Mascarene grey white-eye (Aves: *Zosterops borbonicus*)

**DOI:** 10.1186/1471-2148-10-158

**Published:** 2010-05-26

**Authors:** Borja Milá, Ben H Warren, Philipp Heeb, Christophe Thébaud

**Affiliations:** 1 Museo Nacional de Ciencias Naturales, Consejo Superior de Investigaciones Científicas (CSIC), José Gutiérrez Abascal 2, Madrid E-28006, Spain; 2 UMR PVBMT, Université de La Réunion, 7 chemin de l'IRAT, F-97410 Saint Pierre, Réunion, France; 3 Laboratoire Évolution et Diversité Biologique (EDB), UMR 5174 Centre National de la Recherche Scientifique (CNRS) - Université Paul Sabatier, 118 Route de Narbonne, F-31062 Toulouse, France

## Abstract

**Background:**

Oceanic islands provide unique scenarios for studying the roles of geography and ecology in driving population divergence and speciation. Assessing the relative importance of selective and neutral factors in driving population divergence is central to understanding how such divergence may lead to speciation in small oceanic islands, where opportunities for gene flow and population mixing are potentially high. Here we report a case of genetic and morphological structure in the Mascarene grey white-eye (*Zosterops borbonicus*) a species that shows a striking, geographically structured plumage polymorphism on the topographically and ecologically complex island of Réunion, yet is monotypic on the relatively uniform neighbouring island of Mauritius.

**Results:**

Analysis of 276 AFLP loci in 197 individuals revealed prolonged independent evolution of Réunion and Mauritius populations, which is congruent with previous mtDNA assessments. Furthermore, populations on Réunion showed significant differentiation into three main genetic groups separating lowland from highland areas despite the small geographic distances involved. Genetic differentiation along the altitudinal gradient is consistent with morphometric analysis of fitness-related traits. Birds in the highlands were larger, yet had relatively smaller beaks than in the lowlands, suggesting the role of selection in shaping morphology and restricting gene flow along the gradient. No genetic differentiation between plumage morphs was detected in neutral markers, suggesting that plumage differences are of recent origin.

**Conclusions:**

Our results suggest a dual role of vicariance and natural selection in differentiating populations of a passerine bird in an oceanic island at very small spatial scales. We propose a combination of past microallopatry driven by volcanic activity and selection-constrained dispersal along steep ecological gradients to explain the striking levels of population structure found within the island, although the possibility that genetic differences evolved *in situ *along the gradient cannot be ruled out at present. The lack of congruence between genetic groups and plumage morphs suggests that the latter are of recent origin and likely due to social or sexual selection acting on few loci. The presence of sharp and stable contact zones between plumage morphs suggests that they could be on independent evolutionary trajectories, yet whether or not they represent incipient species will require further research to directly assess the degree of reproductive isolation among them.

## Background

Island archipelagoes have played a crucial role in the diversification of biotas, and oceanic islands have long been recognized as natural laboratories for the study of evolutionary processes [1-4]. Geographically isolated populations of a colonist lineage on different islands often diverge, leading to the accumulation of species on individual islands within an archipelago [5-7]. Furthermore, different selection pressures acting on populations within a single island can promote intra-island speciation, yet this depends mostly upon the spatial scale of both intraspecific gene flow and divergent natural selection pressures relative to the size of the island [8,9]. Thus, for a given colonist lineage, intra-island speciation should contribute to an increase in species richness within an archipelago only if some islands are large enough to prevent the homogenizing effect of gene flow, or divergent selection pressures are sufficiently strong to drive speciation in spite of gene flow. While geographic isolation has long been recognized as a key factor in speciation [1,10], it appears that intra-island speciation can take place only when island size exceeds a clade-specific threshold [11]. Below that threshold, small islands are thought to simply lack opportunities for geographic isolation or spatially heterogeneous selection [12,13]. In birds, several recent studies have emphasized the lack of examples of intra-island speciation on islands smaller than Madagascar, with all cases of related endemic species within an island being best explained by multiple invasions [4,9,14,15]. Yet evidence of evolutionary divergence in bird populations within small islands exists [16,17], suggesting that population differentiation is possible under strong selection despite gene flow and may lead to speciation if historical events combine with ecological differentiation to generate strong ecogeographic barriers. Because of their unique characteristics as "closed systems", cases of within-island population structure provide unique opportunities to investigate the relative roles of ecological and allopatric factors in driving differentiation, as well as the magnitude and spatial scale at which gene flow precludes divergence.

**Figure 1:**
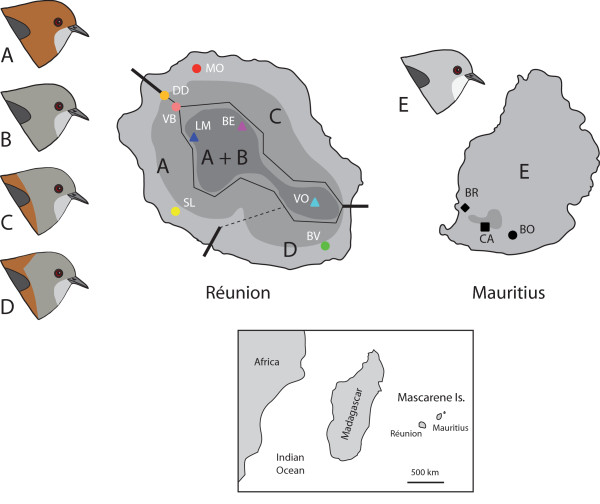
**Sampling localities and geographic distribution of *Z. borbonicus *plumage morphs in Réunion and Mauritius, the two largest Mascarene Islands**. Light, medium and dark grey layers on maps correspond to elevations 0-600 m, 600-1000 m, and 1000-3000 m, respectively. Thick lines correspond to sharp contact zones between morphs with narrow hybrid zones at rivers or lava flows. Thin black lines represent approximate boundaries between morphs away from contact zones (where considerable intergradation occurs). Dashed line indicates inferred boundary in unsurveyed area. Plumage morphs are coded as follows: A = brown, B = grey, C = grey-headed brown, D = grey-headed brown nape, E = Mauritius grey.

Here we examine patterns of genetic and morphological differentiation in the Mascarene grey white-eye endemic to Réunion (*Zosterops b. borbonicus*) and Mauritius (*Z. b. mauritianus*) in the Mascarene Islands, some 800 km off the coast of Madagascar in the southern Indian Ocean [18]. A single light-grey plumage morph is found on the relatively old and flat island of Mauritius (1,865 km^2^), yet the species is composed of four distinct plumage morphs on the topographically and ecologically complex island of Réunion (2,512 km^2^). The colour morphs on Réunion were first described as different species or as individual, age or sex variants [19-21], and then as four endemic subspecies [22-24]. The morphs are geographically structured and separated by either sharp contact zones at rivers and lava flows, or by less defined altitudinal clines of colouration (Fig. 1), a unique pattern within a bird species on a small oceanic island. The grey morph is restricted to the highland areas (typically > 1000 m), where it is sympatric with the all brown morph. The latter is also found on the west side of the island down to sea level (where it is lighter in colour). The all brown morph comes into contact with the grey-headed brown morph of the eastern side of the island at the Galets River, and with the grey-headed brown-nape morph from the south end of the island at the St. Etienne River. Our field sampling since 2007 revealed that the geographic structure of colour morphs (Table 1) and the position of contact zones separating them has remained unchanged since first described by Gill [23] over 40 years ago, suggesting that some mechanisms maintain the distinctiveness of the morphs in spite of the birds' high vagility.

**Table 1:** Sampling sites, elevation, and colour morph frequencies per locality.

Locality (code)	*n*	Elevation (m)	Colour morph
Réunion Island			
Dos d'Ane (DD)	15	775	C (5), U (10)
Roche Verre Bouteille (VB)	6	1,251	B (1), C (2), U (3)
Moka (MO)	21	219	C (21)
Bélouve (BE)	31	1,518	A (31)
Volcano (VO)	43	2,246	A (31), B (9), U (3)
Le Maido (LM)	27	2,062	A (7), B (18), U (2)
Basse Vallée (BV)	19	687	D (19)
St. Leu (SL)	20	509	A (20)
Mauritius Island			
Black River (BR)	5	10	E (5)
Camp Station (CA)	29	600	E (29)
Bel Ombre Station (BO)	23	278	E (23)

A = brown, B = grey, C = grey-headed brown, D = grey-headed brown nape, E = Mauritius grey, U = uncertain. The latter category includes individuals with mixed brown and grey plumage, representing either juvenal plumage or potential inter-morph hybrids. See Fig. 1 for depictions of plumage morphs.

Analyses of mitochondrial DNA sequences revealed a deep phylogenetic break between reciprocally monophyletic populations of Réunion and Mauritius [25], and an estimated coalescence time to a common ancestor within the last 430,000 years. As shown by Warren et al. [25], *Z. borbonicus *forms a monophyletic clade that is sister to the divergent *Z. olivaceus *clade (also found on both Réunion and Mauritius). The divergence of these two clades (i.e. all four Mascarene forms) along with *Z. mouroniensis *and *Z. semiflavus *(from the Comoros and Seychelles Islands, respectively) from their closest continental relatives occurs deep within the Zosteropidae phylogeny and substantially pre-dates the divergence of all other western Indian Ocean *Zosterops *from their closest continental relative. Therefore, while multiple hypotheses for the direction of inter-island *Zosterops *colonization in the Mascarenes are plausible, based on mtDNA data and the sampling of all known living and extinct western Indian Ocean island forms, the Réunion population of *Z. borbonicus *can only result from a single colonization of the island and long-term divergence from the Mauritian population. This leaves open the possibility of an intra-island evolutionary radiation on Reunion.

While initial divergence could have resulted from drift or divergent selection in geographically separate areas within the island, three lines of evidence suggest the role of assortative mating in maintaining Réunion morphs separated: (1) The birds are good flyers and the geographic barriers separating the morphs are very narrow (0.3-5 km) relative to the birds' dispersal ability; (2) although intermediates occur, most sympatric grey and brown individuals in the highlands are easily assigned to either morph despite forming highly-social, mixed foraging groups; and (3) putative hybrids can be easily identified by a clear mixing of brown and grey colour, and are rare in nature [23]. Given this evidence for at least incipient reproductive isolation among *Z. borbonicus *morphs within Réunion, we carried out a survey of genome-wide genetic variation using amplified fragment length polymorphism (AFLP) to investigate inter-island divergence as well as the relationship between any genetic structure of populations within Réunion and the geographic distribution of colour morphs, geographic barriers, and ecological gradients. We also analyzed variation in fitness-related morphological traits to assess the role of selection in driving phenotypic divergence.

## Results

### Morphological variation

**Figure 2:**
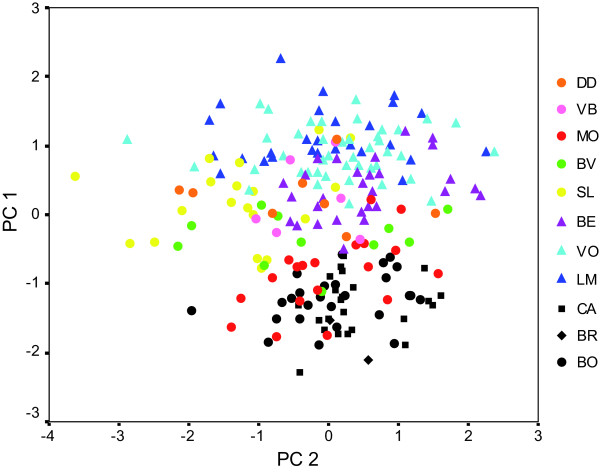
**Principal components analysis of morphological variation**. Based on 6 morphological traits (wing length, tail length, tarsus length, bill length, bill width and bill depth) across 214 individuals of *Z. borbonicus *from 11 localities in Réunion (colored markers, with highland sites represented by triangles and lowland sites by filled circles) and Mauritius (black markers). The variance explained by PC1 and PC2 is 44% and 27%, respectively (see Table 2)

**Figure 3:**
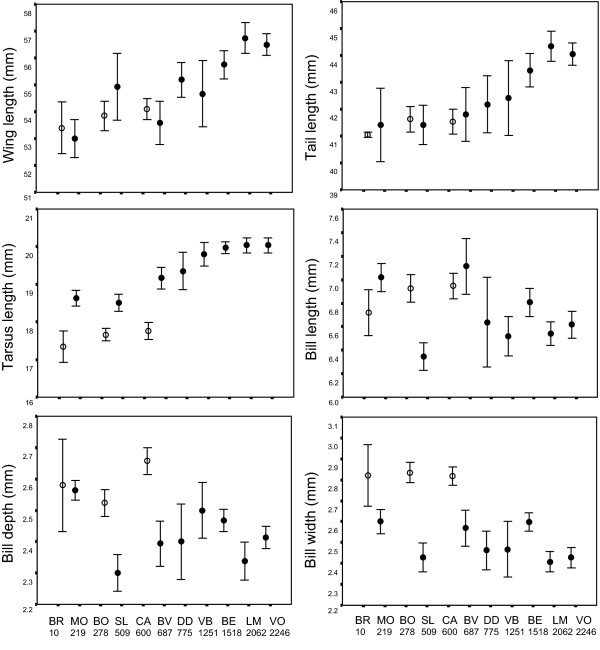
**Morphological trait means per site**. Sites are coded as in Table 1 and arranged from left to right according to elevation, with altitude (in meters) shown under each code. Bars around means represent 95% confidence intervals. Empty markers correspond to localities in Mauritius, full markers represent localities in Réunion.

A principal components analysis (PCA) revealed the separation of individuals along PC1 into two main clusters corresponding mainly to lowland (< 1000 m) and highland (>1000 m) populations (Fig. 2). The PC1 axis represents size, with high positive factor loadings for wing, tail and tarsus lengths, and negative values for bill length, width and depth (Table 2). Birds in the highlands are therefore characterized by longer wings, tails and tarsi, yet smaller beaks than birds in the lowlands. Birds from Mauritius had significantly shorter tarsi and narrower bills than Réunion birds (Fig. 3), yet overall clustered with lowland Réunion birds from the eastern side of the island in the multivariate analysis (Fig. 2).

**Table 2:** Principal components analysis of morphological data.

Variables	Factor loadings	
	PC1	PC2
Wing length	.71	.48
Tail length	.67	.55
Tarsus length	.83	.23
Bill length	-.37	.69
Bill depth	-.57	.63
Bill width	-.74	.40
Variance explained	44%	28%

Coefficients of correlation between morphological variables and the derived principal components extracted by principal components analysis.

Wing, tail and tarsus length means per site showed a strong positive correlation with elevation, whereas bill measurements showed a weak, inverse pattern (Fig. 3). According to a linear regression analysis, elevation was a good predictor of tarsus length (R^2 ^= 0.512, *F*_1,230 _= 240.931, *P *< 0.0001), wing length (R^2 ^= 0.292, *F*_1,229 _= 94.233, *P *< 0.0001) and tail length (R^2 ^= 0.288, *F*_1,211 _= 85.282, *P *< 0.0001), yet explained less of the variance in bill length (R^2 ^= 0.048, *F*_1,231 _= 11.736, *P *= 0.001), bill depth (R^2 ^= 0.095, *F*_1,231 _= 24.131, *P *< 0.0001), and bill width (R^2 ^= 0.222, *F*_1,231 _= 65.784, *P *< 0.0001).

### Genetic diversity and structure

The four pairs of selective primers used to generate AFLP markers yielded a total of 276 scorable loci, out of which 63 (23%) were polymorphic (Table 3). The proportion of variable loci per primer pair ranged from 16 to 39%, which is within the range of values found in birds and other vertebrates [26]. However, the difference in genetic diversity between islands was marked, with Réunion showing more than twice the percent of polymorphic loci (92% vs. 40%) and expected heterozygosity (0.237 vs. 0.118) than Mauritius (Table 4).

**Table 3:** Primers used to generate AFLP profiles and variability of loci.

Combo	E primer	M primer	Total scorable loci	Mono- morphic loci	Variable loci	% Variable
C1	E-tct	M-cga	41	25	16	39
C2	E-tct	M-ctt	43	33	10	23
C3	E-tag	M-cag	95	80	15	16
C4	E-tgc	M-cat	97	70	22	23
Total			276	213	63	23

**Table 4:** Genetic diversity estimated from 62 variable AFLP loci.

Locality	n	No. alleles^a^	**No. alleles (freq. > 5%)**^**b**^	**% polymorphic loci**^**c**^	**No. private alleles**^**d**^	**He**_**e**_^**e**^	**SE (H**_**e**_**)**
Réunion:	144	58	50	92	27	0.237	0.023
DD	7	44	44	60	0	0.206	0.026
VB	6	36	36	36	0	0.120	0.023
MO	16	41	41	55	0	0.198	0.026
BE	24	43	41	58	0	0.204	0.026
VO	31	40	39	55	0	0.214	0.027
BV	16	43	43	58	1	0.176	0.025
SL	19	45	45	63	0	0.203	0.025
LM	25	52	48	71	2	0.190	0.023
Mauritius:	53	35	34	40	4	0.118	0.023
BR	5	33	30	35	0	0.111	0.023
CA	26	29	29	23	0	0.079	0.021
BO	22	32	31	32	0	0.111	0.023

Allele frequencies are calculated according to the method by Lynch and Milligan [58]. ^a ^Total number of alleles per locality. ^b ^Number of alleles with frequency greater than 5%. ^c ^Percentage of loci (of frequency > 5%) that are polymorphic out of the total 62 loci. ^d ^Number of alleles unique to a single population. ^e ^Mean expected heterozygosity. See Table 1 for locality names.

**Figure 4:**
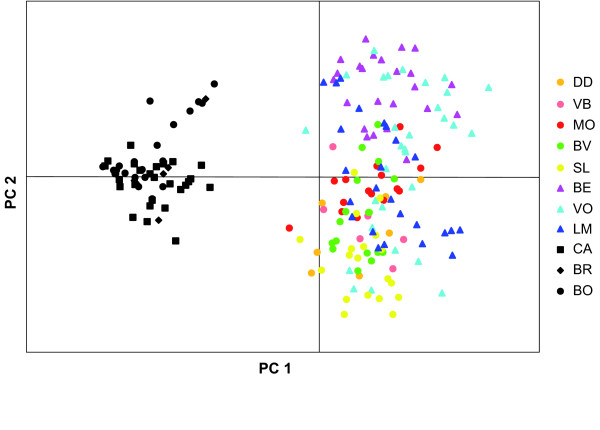
**Principal coordinate analysis of genetic variation**. Based on 63 AFLP loci across 197 individuals of *Z. borbonicus *from 11 localities in Réunion (colored markers, with highland sites represented by triangles and lowland sites by filled circles) and Mauritius (black markers). The variance explained by PC1 and PC2 is 40% and 16%, respectively. Site codes as in Table 1.

Differentiation between the two islands accounted for almost one third of the variance in genetic variation, with structuring among populations within islands and within-population variation explaining 10% and 63% of the variance, respectively (Table 5). A plot of the first two coordinates (55% cumulative variance explained) from a principal coordinate analysis (PCO) of AFLP variation revealed a clear separation between Réunion and Mauritian localities along the first coordinate (40% of the variance) (Fig. 4). No structure was apparent within Mauritius, although sampling there was limited and restricted to the southern part of the island. However, clear separation of populations was apparent within Réunion, with some highland sites appearing differentiated from lowland sites (e. g., Bélouve and St. Leu) along the first principal coordinate (Fig. 4).

**Table 5:** Analysis of molecular variance of AFLP variation.

Variance component	df	SS	Est. Variance	% Variance	Value	*P*
Among islands (Φ_RT_)	1	208.30	2.36	27%	Φ_RT _= 0.268	0.001
Among pops. within islands (Φ_PR_)	9	187.44	0.90	10%	Φ_PR _= 0.139	0.001
Within pops. (Φ_PT_)	186	1032.37	5.55	63%	Φ_PT _= 0.370	0.001
Total	196	1428.10	8.81			

*Z. borbonicus *individuals from 11 localities grouped into two groups corresponding to Réunion and Mauritius.

**Figure 5:**
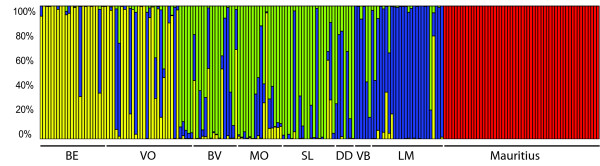
**Spatial genetic structure according to a Bayesian assignment probability analysis using the program STRUCTURE 2.2**. Each vertical bar represents an individual, with each colour corresponding to the posterior probability of assignment to each of an optimal number of 4 clusters, represented by different colours.

A Bayesian population assignment analysis using STRUCTURE 2.2 corroborated the marked separation of Mauritius with respect to Réunion. An optimal K of 4 revealed a Mauritian cluster and three additional clusters within Réunion (Fig. 5). Separate analysis for each island did not change these results, yielding optimal K values of 3 and 1 for Réunion and Mauritius, respectively. The three clusters identified within Réunion are geographically structured and correspond roughly to lowland areas (green in Fig. 5), western highlands (blue) and eastern highlands (yellow), respectively. Several individuals within each of these main areas clustered with other groups, suggesting either ongoing movement of individuals or past events of gene flow. For example, 5 individuals from the Volcano site (VO in Fig. 1) showed high assignment probability to the green lowland cluster. Interestingly, and despite the relatively low sample sizes, individuals from locality VB had high probabilities of assignment to the blue cluster, whereas in locality DD, just 1 km downhill from VB (see Fig. 1), the probability of belonging to the green cluster was higher. Overall, our results suggest that population structuring within Réunion occurs at a very small spatial scale.

*F_ST _*values between islands were high (Table 6), with an average Φ_PT _of 0.38 (SD = 0.06). Within Réunion, differentiation between populations was remarkably high, with an average pairwise Φ_PT _of 0.148, and highest values were obtained in highland-lowland comparisons. A Mantel test of the correlation between a matrix of geographic distances (Table 6) and *F_ST _*values among localities was not significant (*P *= 0.629), suggesting that factors other than mere isolation-by-distance are responsible for the observed genetic structure.

**Table 6:** Matrices of genetic and geographic distances among *Z. borbonicus *localities.

	DD	VB	MO	BE	VO	BV	SL	LM	CA	BR	BO
**DD**		3.4	16.4	20.2	42.9	53.9	19.3	10.9	226.0	219.2	221.1
**VB**	0.148		14.1	16.8	39.6	50.9	19.5	9.6	223.5	216.7	218.5
**MO**	0.093	0.220		14.9	36.7	50.0	32.5	21.5	209.6	202.8	204.7
**BE**	0.191	0.272	0.174		23.4	35.8	26.5	16.6	212.1	205.6	206.6
**VO**	0.137	0.196	0.112	0.106		13.9	41.7	36.0	204.7	198.8	198.2
**BV**	0.063	0.181	0.168	0.176	0.117		48.5	45.5	209.3	203.8	202.2
**SL**	0.038	0.169	0.146	0.219	0.120	0.057		11.1	238.6	232.1	233.1
**LM**	0.096	0.125	0.175	0.186	0.138	0.139	0.168		228.2	221.6	222.8
**CA**	0.375	0.472	0.409	0.420	0.397	0.369	0.360	0.388		8.3	10.7
**BR**	0.262	0.421	0.336	0.322	0.304	0.274	0.263	0.295	0		14.2
**BO**	0.414	0.500	0.413	0.410	0.396	0.402	0.388	0.402	0.035	0	

Pairwise values of Φ_PT _among localities (below diagonal), and geographic distances among sites in km (above diagonal). All Φ_PT _values above zero are significant at the 0.01 level, except for DD vs. SL, which had a *P *value of 0.07. Mauritian localities are CA, BR and BO.

**Figure 6:**
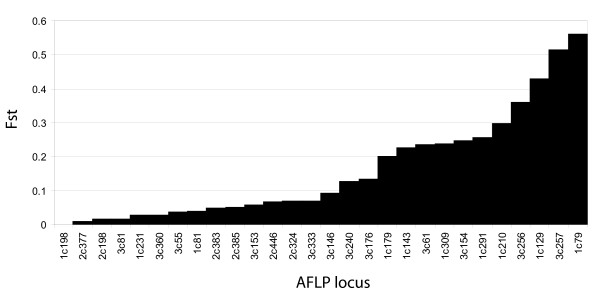
**Pairwise *F_ST _*values per AFLP locus**. Comparison between sites SL (509 m) and LM (2062 m), located on the same slope in western Réunion.

To examine the potential role of selection in driving the marked genetic differentiation between highland and lowland populations, we calculated *F_ST _*values per locus between Le Maido (2,062 m) and St. Leu (509 m) on the east side of the island. A distribution of *F_ST _*values shows that differentiation is not due to just a few loci but to the contribution of many different ones, with 13 out of 44 loci (30%) showing *F_ST _*values > 0.1 (Fig. 6). None of these loci were significant outliers in a distribution of *F_ST _*against heterozygosity, indicating that they are not likely to be under selection [27].

## Discussion

### Geographic differentiation of *Z. borbonicus *in the Mascarenes

Genetic and morphological data examined here revealed a high degree of divergence between *Zosterops borbonicus *populations in Mauritius and Réunion, suggesting prolonged independent evolutionary history with little or no gene flow for a very long time, as well as high levels of structure within the island of Réunion. Inter-island differentiation in both mitochondrial DNA [25] and genome-wide AFLP loci is extensive, suggesting that diversification on Réunion has likely evolved *in situ *and is the product of a single colonization. Indeed, given additional diagnostic characters in plumage and morphology, the two populations of *Zosterops borbonicus *might in fact constitute two separate species [28].

The remarkable degree of differentiation in both AFLP markers and morphological traits within Réunion, reveals very low dispersal rates in *Z. borbonicus *and a strong role for neutral and selective factors in promoting divergence despite the island's small size. The pattern of genetic structure identified with our AFLP data does not correspond to the current distribution of the four plumage morphs on the island, suggesting that differences in plumage colour are of relatively recent origin. Given that the plumage traits are likely controlled by relatively few genes [23], and that only a small fraction of the genome was surveyed with our AFLP markers, the lack of congruence is not surprising and more exhaustive genome-wide surveying and examination of pigmentation-related candidate genes (currently underway) will be needed to identify those loci.

The current pattern of geographic variation of *Z. borbonicus *on the island as revealed by our different datasets is likely the product of four main evolutionary processes: (1) microallopatric isolation of ancestral populations into at least three disjunct demographic units, which has resulted in divergence in neutral genomic markers; (2) morphological differentiation along altitudinal gradients through local adaptation; (3) the appearance of distinct plumage morphs which now exist (or co-exist) in different parts of the island; and (4) the maintenance of these patterns in place over time, through a combination of restricted dispersal and pre and/or post-zygotic mechanisms of reproductive isolation. The combined effect of these different processes has likely given rise to the patterns of divergence and structure observed on Réunion, which are remarkable given the small geographic scale of the island and thus the opportunity for high levels of homogenizing gene flow in a highly vagile organism.

Although the establishment of isolated allopatric populations of birds in a small island is generally unlikely, the inferred pattern of microallopatry responsible for the genetic structure found in neutral markers could be tied to the active eruptive history of Réunion's volcanoes, the "Piton des Neiges" on the northwest, and the "Piton de la Fournaise" in the southeast. Whereas the island of Mauritius is about 8 million years old [29], the emergence of lava above sea level in Réunion was around 2.1 Mya [30], and was followed by two periods of frequent explosive eruptions, one between 0.35 and 0.13 Mya (after which the center of the volcano collapsed giving rise to the three large calderas seen today), and one between 0.05 and 0.012 Mya [30,31]. In turn, the still active "Piton de la Fournaise", which originated about 0.55 Mya, has had three main eruptive phases from 0.53 to 0.29 Mya, from 0.22 to 0.05 Mya, and from 0.013 to the present [32]. Given the combined activity of both volcanoes, barren lava fields have covered most of the island at different times over the last 350,000 years. For at least part of this period, areas with suitable vegetation for the maintenance of *Z. borbonicus *populations were probably restricted to isolated patches. The presence of small areas (10 to 100 km2) with original basaltic shields at ground level in areas scattered around Réunion (see Fig. 1 in Deniel *et al*. 1992) suggests that these areas have remained free of lava for hundreds of thousands of years, and thus could have harboured forested areas capable of sustaining bird populations. Since the time of the last eruption of the "Piton des Neiges", as recent as 12,000 years ago, forested areas would have expanded throughout the island, bringing differentiated *Z. borbonicus *populations into contact. Given the very limited rates of dispersal inferred from our genetic and morphological results, and the apparent reluctance to cross barren areas such as river beds, large lava flows must have represented formidable barriers to dispersal among patches across the island.

The lack of congruence between the pattern of neutral structure found and the distribution of color morphs suggests that the latter evolved at a later stage and could indeed be of recent origin, as changes in plumage pigmentation can be fast in birds [33-35].

### The relative roles of drift and selection in restricting gene flow and driving divergence

Phenotypic evolution on island populations can be fast under strong natural selection [3,9,16,36-38]. In *Z. borbonicus*, the altitudinal increase of size-related traits like wing, tail and tarsus length, combined with an opposite trend for diet-related traits like beak dimensions, suggests the role of natural selection in driving morphological divergence along steep altitudinal gradients, as has been documented in other similar avian systems [39-41]. Rapid morphological evolution has been documented in other *Zosterops *species [42,43], yet adaptive changes in morphology can often evolve too fast for neutral genetic markers to show congruent patterns of differentiation [40]. Therefore, the congruent morphological and genetic differentiation between lowland and highland individuals in *Z. borbonicus *(best illustrated by the comparison of St. Leu [509 m] and Le Maido [2,062 m], separated by only 11 km on the west slope of Réunion), poses the question of whether genetic divergence represents *in situ *differentiation through isolation by adaptation (in the absence of geographic isolation: [44,45], or rather a case of secondary contact between groups previously differentiated in allopatry. The fact that as many as 30% of polymorphic loci among these two localities show *F_ST _*values greater than 0.1, and that no loci were found to be under selection according to outlier analysis, supports the hypothesis of secondary contact between cryptic brown morphs rather than parapatric differentiation within a single brown morph. Although more intensive sampling along the island's gradients (currently underway) will be necessary to investigate this question, the morphological results suggest that selection may be an important factor in restricting gene flow up and down the slope, and thus may be important in at least maintaining this marked genetic break despite the short geographic distances involved.

### Zosterops as "great speciators"

Our results are in line with previous work documenting the high diversification capacity of white-eyes (Zosteropidae) [25,46]. Members of the group are successful colonizers due to their high dispersal capacity, which enables them to reach remote islands, yet appear to quickly evolve shifts in dispersal ability that reduce gene flow and promote diversification. This so-called "great speciator paradox" [47] is clearly illustrated by *Z. borbonicus *in the Mascarenes, where upon colonization, and despite the species' good flying capacity, dispersal rate has been dramatically reduced. The evolution of "behavioral flightlessness" has been documented in other members of the genus [43,48,49], and together with sociality, rapid morphological evolution, generalist ecology and short generation time, contribute to the high diversification rates observed in this group [42,46]. Indeed, the relatively high levels of genetic diversity found in AFLP could be attributed to the high levels of sociality observed in *Z. borbonicus *[50], which increases the probability of group colonization and reduces the chance of founder events [51,52].

## Conclusions

Our results shed light on some of the evolutionary mechanisms underlying an extreme case of small-scale intra-island diversification in a passerine bird. The presence of three genetic clusters on Réunion island as revealed by neutral AFLP markers suggests the role of geography in differentiating populations at some time in the past despite the island's small size. In turn, morphometric data suggest the role of ecology-driven selection in differentiating populations and restricting gene flow across habitat types and geographic barriers. However, the fact that patterns of neutral genetic divergence are not geographically congruent with the distribution of plumage morphs, suggests that in addition to genetic drift and natural selection, social or sexual selection may have played a role in driving recent plumage divergence. Future research will be necessary to determine the degree of reproductive isolation between morphs, and thus the extent to which morphs represent incipient species. Thus, whether diversification in Réunion's *Z. borbonicus *is ongoing and represents a case of incipient speciation, or instead the process has been truncated by widespread gene flow upon secondary contact among morphs, remains to be addressed. Previous work on *Zosterops lateralis *in Australia has shown that selection can act upon colonization and then remain static for thousands of years [36]. However, maintenance of narrow hybrid zones between colour morphs and the sympatry of grey and brown morphs in the highlands suggest that reproductive isolating mechanisms are at play. Our results provide a first attempt at unraveling the doubtlessly complex combination of neutral and selective factors that underlie the evolutionary history of the species. Further research along hybrid zones and areas of plumage intergradation will be necessary to better understand this striking case of intra-island diversification.

## Methods

### Field procedures and morphological analysis

Birds were captured using standard mist nets at 11 sampling localities, eight on Réunion and three on Mauritius (Fig. 1, Table 1). Individuals were aged using plumage characteristics, eye and gape color, and the degree of skull ossification [53]. Individuals were weighed and marked permanently with a uniquely numbered aluminum ring. A wing ruler was used to measure the unflattened wing chord (the distance from the carpal joint to the tip of the longest primary) to the nearest 0.5 mm. Dial calipers of 0.1-mm precision were used to measure tail length (from the uropygial gland to the tip of the longest rectrix), tarsus length (from the intertarsal joint to the most distal undivided scute on the tarsometatarsus), bill length (from the anterior end of the nares to the tip of the upper mandible), and bill width and depth (both measured at the anterior end of the nares). All measurements were taken by BM. Manipulation of live birds for ringing, measurements and blood sampling were done under a ringing permit issued by CRBPO Museum National d'Histoire Naturelle (Paris, France), and in compliance with ethical guidelines at Université Paul Sabatier. A special authorization to work on protected species was issued by Direction Régionale de l'Environnement (DIREN Réunion).

We conducted a principal components analysis (PCA) on the correlation matrix of ln-transformed morphological variables. Based on results from the multivariate analysis, we analysed variation in morphological traits along the altitudinal gradient by means of regression analysis, with elevation as the predictor variable. Variables were transformed prior to analyses to meet model assumptions. Assumptions such as normality of the residuals and homogeneity of variances were met.

Since there is no sexual dimorphism in plumage and morphology [23], both males and females were included in the analysis. Individuals with clear juvenile characteristics (orange-grey eye instead of reddish brown, yellow gape, natal plumage, etc.), were excluded from the analysis. However, because the first post-natal moult is complete in this species (juveniles change all feathers, including flight feathers [pers. obs.]), many first and second-year birds could not be distinguished from adults, and thus some young birds might be part of the sample.

### AFLP analysis

Amplified fragment length polymorphism (AFLP) profiles were generated using a protocol modified slightly from Vos *et al*. [54], as detailed in Milá *et al*. [55]. Briefly, whole genomic DNA was digested with restriction enzymes *Eco*RI and *Mse*I (Tru9) and fragments were ligated to oligonucleotide adapters with T4 DNA ligase. A random sub-sample of all restriction fragments was obtained through a pre-selective amplification using primers E-t and M-c, followed by four selective amplifications using primer pairs E-tct/M-cga, E-tcta/M-ctt, E-tag/M-cag, and E-tgc/M-cat (Table 3), with each E primer fluorescently labeled with either VIC or 6FAM fluorescent dyes. Twelve pairs of selective amplification primers were tested with a subset of individuals, but only those producing repeatable and unambiguously scorable profiles were used in the analysis. Selectively amplified fragments were run in an ABI 3700 genetic analyzer with a LIZ500 size standard. Peaks were visualized using GENEMAPPER 3.7 and scored manually. Only unambiguously scorable loci and individuals were included in the analysis and peaks found in less than 2% of individuals were excluded. Methodological error rate was assessed by running a subset of 5 individuals twice from the pre-selective amplification step. The average per-locus error rate for the AFLP data, measured as recommended by Bonin *et al*. [56], was 2.1%.

We estimated allelic frequencies using Zhivotovsky's [57] Bayesian method with uniform prior distributions and assuming Hardy-Weinberg genotypic proportions. Genetic diversity (H_e_), and per-locus *F_ST _*values based on allele frequencies where calculated using the method by Lynch and Milligan [58] as implemented in the program AFLP-SURV v. 1.0 [59]. A matrix of pairwise population *F_ST _*values using the *F_ST _*analogue Φ_PT _was calculated with GENALEX 6.0 [60]. Φ_PT _was calculated as *V*_AP_/(*V*_AP _+ *V*_WP_) where *V*_AP _is the variance among populations and *V*_WP _is the variance within populations. Probability values of pairwise Φ_PT _were based on 9999 permutations.

To assess genetic structure among samples we conducted a principal coordinate analysis (PCO; Orloci [61]) on a genetic distance matrix generated from the binary presence-absence matrix as implemented in GENALEX 6.0. We also examined patterns of population structure using the Bayesian assignment probability test in the program STRUCTURE 2.2 [62]. This program uses a Bayesian approach to generate posterior probabilities of assignment of individuals to each of a given number of populations (K) independently of the geographic locality of origin. As recommended for dominant markers, we applied a model of no admixture with correlated allele frequencies [63] and the optimal value of K was calculated following the method by Evanno *et al*. [64].

To examine the pattern of isolation by distance we tested the correlation between a matrix of pairwise *F_ST _*/(1-*F_ST_*) values among localities and a matrix of the natural log of Euclidean geographic distances between localities. We used a Mantel test with 10,000 permutations of Spearman's rank correlation coefficient with a one-tailed exact test, as implemented in the program ISOLDE in the package GENEPOP[65].

To assess the potential role of selection in driving genetic differentiation among populations, we examined plots of *F_ST _*against heterozygosity under the assumption of Hardy-Weinberg equilibrium to identify significant outlier loci with the program DFDIST[27]. Significance values at the 95% level for outlier loci were obtained by generating a null distribution of *F_ST _*values based on 50,000 simulated loci with a mean *F_ST _*equivalent to the "neutral" mean *F_ST _*of the empirical distribution, which was obtained by trimming the 30% highest and lowest *F_ST _*values [27].

## Authors' contributions

BM, CT and PH designed the study; BM, CT and BW carried out the field work; BM took the morphologic measurements in the field and run the morphometric analyses with input from PH; BM generated the molecular data and conducted all genetic analyses; BM and CT wrote the manuscript with contributions from all authors, who read and approved the final manuscript.
